# Objective Outcomes Evaluation of Innovative Digital Health Curricula. Comment on “Undergraduate Medical Competencies in Digital Health and Curricular Module Development: Mixed Methods Study”

**DOI:** 10.2196/26034

**Published:** 2021-05-28

**Authors:** Alexander Grzeska, Shan Ali, Tomasz Szmuda, Paweł Słoniewski

**Affiliations:** 1 Medical University of Gdansk Gdańsk Poland

**Keywords:** digital health, eHealth, mHealth, digital health education, elective module, eHealth education, curriculum, medical school, digital health mindset, qualitative research, interview, survey

We read with great interest the study by Poncette et al [1] on the development of a curricular module for digital health and undergraduate medical competencies.

The paper [1] illuminates the importance of implementing digital health training for future physicians in the curricula of medical universities. While the paper discusses the benefits of building a critical and experience-based mindset among future physicians, we believe that measuring the performance of and skills acquired by students before and after participation may augment the development and implementation of digital health competencies in the medical curricula.

A survey found high levels of satisfaction among students participating in the elective module. Confidence levels, as well as the acquisition of knowledge and skills in digital health, were reported to be high. However, this analysis is subjective as it is based on the student’s own opinion of their knowledge. In order to obtain more objective results, we recommend the assessment of individual skills by a standardized exam before and after participating in the module. Supplementing the opinion-based survey with exam performance may shed light on the actual perceived level of student competence while reducing selection bias. This would highlight the effectiveness of the course and give medical departments and universities more confidence to adopt digital health competencies in their curricula.

We commend Poncette et al [1] not only for their findings but for how they encourage a more interactive and nonclassical way of teaching. We hope that the high satisfaction among students may encourage medical universities to adopt this new model of teaching. The passive transfer of knowledge has been the dominating system used in medical universities [2] whereas active learning methods such as a “peer-to-peer” [1] teaching approach with an open discussion is rarely found, thus, resulting in high levels of satisfaction [3]. Therefore, we hope that this paper may persuade medical universities to adopt this new teaching approach.

The introduction of digital health competencies into the medical curricula remains key to the success of future physicians as shown by Poncette et al [1]. Therefore, we advocate for more objective student evaluation methods—before and after participating in learning modules—so that traditional medical institutions may be compelled to adopt this new teaching approach.
